# Granger causal time-dependent source connectivity in the somatosensory network

**DOI:** 10.1038/srep10399

**Published:** 2015-05-21

**Authors:** Lin Gao, Linda Sommerlade, Brian Coffman, Tongsheng Zhang, Julia M. Stephen, Dichen Li, Jue Wang, Celso Grebogi, Bjoern Schelter

**Affiliations:** 1 Institute of Biomedical Engineering, Key Laboratory of Biomedical Information Engineering of Education Ministry, Xi’an Jiaotong University, Xi’an 710049, Shaanxi, P. R. China; 2 State Key Laboratory of Manufacturing Systems Engineering, Xi’an Jiaotong University, Xi’an 710049, Shaanxi, P. R. China; 3 Institute for Complex Systems and Mathematical Biology, King’s College, University of Aberdeen, Aberdeen AB24 3UE, UK; 4 The Mind Research Network and Lovelace Biomedical and Environmental Research Institute, Albuquerque, NM 87131, USA; 5 Department of Neurology, University of New Mexico, Albuquerque, NM 87131, USA

## Abstract

Exploration of transient Granger causal interactions in neural sources of electrophysiological activities provides deeper insights into brain information processing mechanisms. However, the underlying neural patterns are confounded by time-dependent dynamics, non-stationarity and observational noise contamination. Here we investigate transient Granger causal interactions using source time-series of somatosensory evoked magnetoencephalographic (MEG) elicited by air puff stimulation of right index finger and recorded using 306-channel MEG from 21 healthy subjects. A new time-varying connectivity approach, combining renormalised partial directed coherence with state space modelling, is employed to estimate fast changing information flow among the sources. Source analysis confirmed that somatosensory evoked MEG was mainly generated from the contralateral primary somatosensory cortex (SI) and bilateral secondary somatosensory cortices (SII). Transient Granger causality shows a serial processing of somatosensory information, 1) from contralateral SI to contralateral SII, 2) from contralateral SI to ipsilateral SII, 3) from contralateral SII to contralateral SI, and 4) from contralateral SII to ipsilateral SII. These results are consistent with established anatomical connectivity between somatosensory regions and previous source modeling results, thereby providing empirical validation of the time-varying connectivity analysis. We argue that the suggested approach provides novel information regarding transient cortical dynamic connectivity, which previous approaches could not assess.

The human brain can be seen as a multi-layered complex network, stimulated by external inputs, to process affective and cognitive information, thus instructing the execution of appropriate responses^1^,^2^,^3^,^4^. The exploration of stimulus-activated neural sources and their connectivity provides deep insights into the brain mechanisms of information processing within the complex brain network^5^,^6^. The human somatosensory system is studied by means of functional magnetic resonance imaging (fMRI), magnetoencephalography (MEG) or electroencephalography (EEG) with tactile sensory stimulation. Unilateral sensory stimulation reliably elicits contralateral somatotopic activation of the primary (SI) and secondary (SII) somatosensory cortex^7^,^8^,^9^,^10^,^11^,^12^. Some studies reported reliable activation of contralateral SI and bilateral SII in response to unilateral sensory stimuli^13^,^14^,^15^,^16^,^17^,^18^,^19^,^20^,^21^, and a few studies observed occasional ipsilateral SI activation^22^,^23^,^24^,^25^,^26^. Despite these numerous reports few studies have investigated the quantitative relationship of the cortical activations, let alone the time-dependent Granger causal connectivity. Thus appropriate investigations remain to date rare and sometimes inconclusive. Consequently the information flow among cortices involved in the somatosensory information processing is not well understood, especially the relationship between the contralateral somatosensory cortices (both SI and SII) and ipsilateral SII^9^,^27^.

In the neurosciences, functional connectivity of neural networks is usually estimated by classical methods, such as correlation and coherence, based on time- or frequency domain analysis in sensor space. These functional connective methods do not provide a direction of information flow. When dealing with brain functional data sets, ultimately the algorithms should be capable of dealing with truly multivariate data accounting for direct and indirect influences. The algorithms also need to cope with various noise sources, address both linear and nonlinear systems, and provide the strengths of the directed interactions for each sampling point. Furthermore, they need to provide a rigorous statistical framework. Until now a rigorous mathematical framework comprising all of these features was still lacking. Some methods could estimate time-dependent connectivity patterns without concern for observational noise and non-stationarity^28^,^29^. Other techniques that enable investigation of directional relationships on very short time scales from multiple realisations of short and transient noisy time series have been proposed^30^,^31^. An overarching framework for data-based modelling, combining state space modelling^32^,^33^,^34^ for autoregressive models with time-dependent autoregressive coefficients, enabling the estimation of time-resolved renormalised partial directed coherence in the frequency domain, fulfills all the above requirements. The approach quantifies the direction and strength of time-dependent network connectivity without relying on any prior assumption about the nonlinear, non-stationary and stochastic brain signals^35^,^36^. The performance of this novel approach has also been validated in a simulation study^35^.

In this paper, we apply the time-varying Granger causal connectivity approach to MEG data from healthy humans. We use reliable unilateral sensory paradigms to explore source connectivity patterns within the human somatosensory system in response to tactile stimulation by identifying time-dependent effective connectivity from high-density MEG recordings. We hypothesize that somatosensory activation, as measured by MEG in humans, should qualitatively and quantitatively reflect the effective connectivity of the somatosensory network. The source locations and single-trial waveforms are estimated by Minimum Norm Estimation (MNE) to mitigate the field spread in MEG recordings, which complicates the interpretation of cortical connectivity patterns at the sensor level. The time-dependent effective connectivity patterns among brain sources is calculated by the new approach combining renormalized partial directed coherence with state space modeling. We also employ a rigorous statistical evaluation procedure to guarantee the applicability to measured signals. This study demonstrates the effectiveness of the time-varying Granger causal connectivity to explore the brain effective connectivity in source space in MEG/EEG fields, which provides a new way to investigate the network interplay between stimulus-evoked cortical activations.

## Results

**Figure 1:**
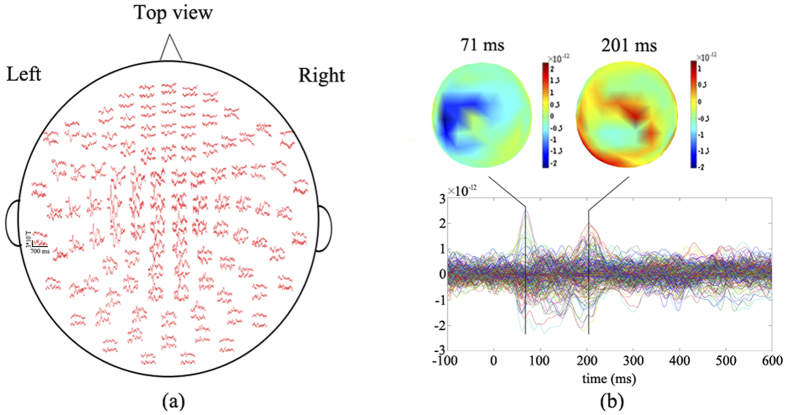
(**a**) Spatial distribution of MEG averaged responses. At each sensor location, the traces illustrate signals recorded by two orthogonal gradiometers. (**b**) Group averaged waveforms from each channel are plotted in different colors and superimposed and scalp topographies at 71 ms and 201 ms peak latencies are displayed.

The spatial distribution of the grand averages for the healthy subjects is illustrated in Fig. 1(a). We found that the evoked response to right index finger stimulation is most prominent in left central channels consistent with activation from postcentral gyrus of the anterior parietal lobe, where primary and secondary somatosensory cortex (SI and SII) are located, and the averages over the other brain areas look like the non-phase locking activity in the background. Figure 1(b) shows the grand average somatosensory MEG waveforms in all channels for the subjects, with the scalp distribution at the latencies of several typical MEG peaks (71 ms and 201 ms). Significant time points around the peak of somatosensory were selected by visual inspection of the waveform. The grand average MEG waveform consisted of a clear positive and negative peak at 71 ms followed by a negative peak at 201 ms. The scalp distribution at 71 ms extended towards temporal regions with negative maximum at the contralateral temporal region and the central region. The scalp topography at 201 ms showed a clear maximum at the ipsilateral central region. According to the results of the source distribution among all the subjects (Table 1), we selected three sources here. The source locations are SI-l (-35 mm, -29 mm, 47 mm), SII-r (41 mm, 1 mm, -8 mm), and SII-l (-41 mm, 1 mm, -8 mm) at the group level. The fitted sources explained the scalp distribution of MEG with RV of 5.5% and 10.8 ± 4.2% at the group level and single-subject level, respectively.

**Figure 2:**
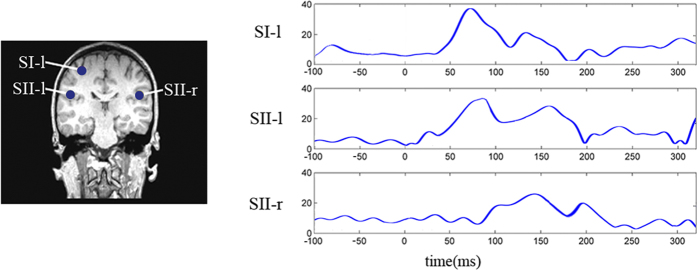
Representative source locations and the corresponding grand average source time courses of somatosensory MEG responses from all participants are presented.

Figure 2 shows the locations and grand average time series of the representative sources (SI-l, SII-r and SII-l) of the subjects. The results indicate that 1 the sources explaining the peak at 71 ms are mainly located in contralateral SI (SI-l) and contralateral SII (SII-l), 2 the sources explaining the peak at 201 ms are mainly located in ipsilateral SII (SII-r). Post hoc comparisons by Fisher’s least significant difference (LSD) test revealed that 1 the peak latencies of SI-l are significantly shorter than the peak latencies of SII-r (P < 0.001), 2 the peak latencies of SII-l are significantly shorter than the peak latencies of SII-r (P < 0.001).

**Figure 3:**
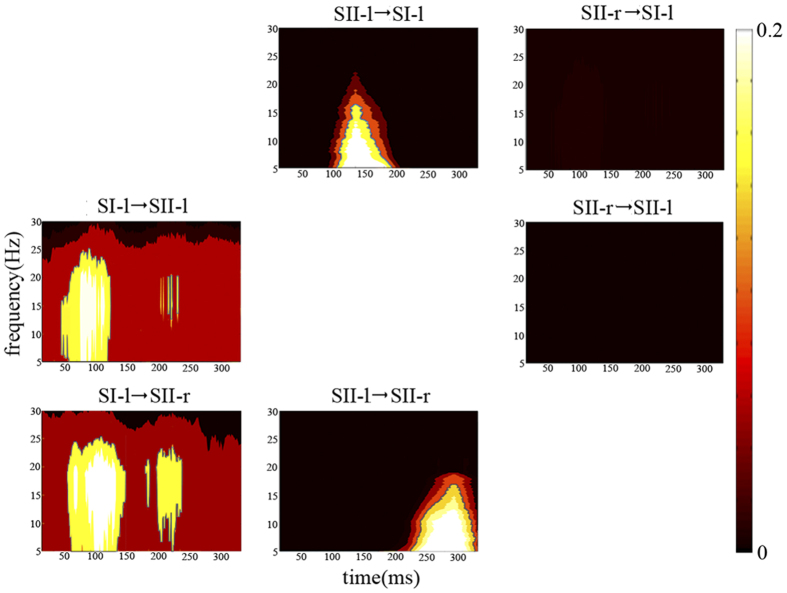
Time-frequency representations of rPDC as a measure of time-dependent Granger causal influences within the neural network between SI-l, SII-l and SII-r averaged across all the subjects. The regions circled by blue lines had significantly larger rPDC values than those in the reference interval from -100 ms to 0 ms (P < 0.01).

**Figure 4:**
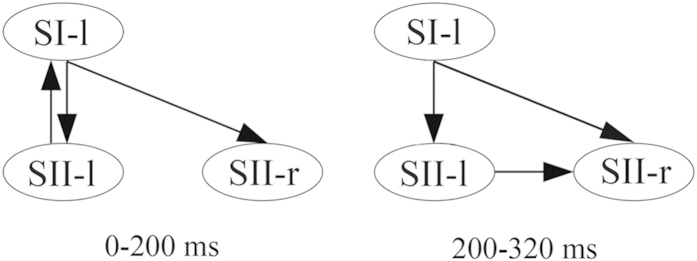
Graph obtained from rPDC analysis applied to source time series located at SI-l, SII-l and SII-r for somatosensory evoked MEG.

Single-trial data of somatosensory source waveforms (SI-l, SII-l and SII-r), including 120 to 180 trials for each source of each subject, obtained from source analysis, were used in the rPDC estimation to assess the relationship among sources. The rPDC order, *p*, selected using Akaike’s Information Criterion (AIC), ranged from 30 to 60; the order was selected for each analysis optimally using AIC. We also projected the data in the reference interval from -100 ms to 0 ms to the representative sources. Time-varying effective connectivity patterns among somatosensory sources for the subjects, represented as time-frequency regions that have significantly increased rPDC values compared with the reference interval (P < 0.01), are summarized into the following temporally distinct groups within the post-stimulus interval around 0-320 ms (Fig. 3). Significant increases in effective connectivity are observed from SI-l to both SII-l (20-230 ms, 5-25 Hz) and SII-r (20-230 ms, 5-25 Hz), from SII-l to SI-l (90-200 ms, 5-17 Hz) and from SII-l to SII-r (220-320 ms, 5-19 Hz). The time-resolved directed network structure can be inferred from Fig. 3 and Table 2 (see also Fig. 4). The cortical information was mainly transmitted from SI-l to SII-l and to SII-r (20-230 ms, the latter one about 5 ms later than the former). Later during the post stimulus interval, the information flow was observed from SII-l to SI-l (90-200 ms) and then to SII-r (220-320 ms).

## Discussion

In this study, we applied a novel source connectivity analysis method based on causality inference to assess the time-varying effective connectivity among stimulus-elicited neural sources for healthy participants. The time-varying effective connectivity revealed that the cortical information flow of somatosensory input at early latencies was processed through a complex pattern of both feedforward and feedback interactions between contralateral SI and bilateral SII: 1) from contralateral SI to contralateral SII, 2) from contralateral SI to ipsilateral SII, 3) from contralateral SII to contralateral SI, and 4) from contralateral SII to ipsilateral SII.

Detection of causal relationships between neural processes is commonly hampered by time-dependent dynamics and observational noise contamination. Time-dependent dynamics is of particular interest in EEG/MEG studies because electrophysiological information is believed to be transferred in part via short bursts of oscillatory brain activity^37^,^38^,^39^. Effective connectivity analysis based on the concept of Granger causality^40^ allows for the evaluation of the direction and strength of causality between neuronal activations, e.g., PDC^41^. However, Granger causality cannot reveal the dynamic effective connectivity for transient and markedly nonstationary neurophysiological processes^42^. In order to address these challenges, we employed a combination of renormalized partial directed coherence and non-linear state space modeling allowing for the detection of time-dependent and eventually continuously changing causal influences in multivariate processes without relying on prior assumptions about the nonlinear, non-stationary and stochastic brain signals^36^. Schelter *et al.* demonstrated its performance in a simulation study and proved that it combines characteristics that others lacked so far^35^. In the present study, we used this novel approach to explore the causal influence empirically by testing the performance of the method with somatosensory evoked MEG responses of healthy subjects.

In neuroscience studies, the event-related experiment with different stimulation paradigms is a fundamental method to activate corresponding brain areas connected by underlying neuronal networks. The activated network is characterized by the evoked data in epochs, which are mixed with field spread. The field spread often complicates the interpretation of cortical connectivity patterns at the sensor level^5^. Carrying out connectivity analysis in source space is the best choice to disentangle the above problem, providing direct estimation of the interaction between neuronal sources and attenuating the effect of field spread^5^,^44^. As expected, we observed that somatosensory sources were mainly located at contralateral SI and bilateral SII after right index finger somatosensory stimulation when applying CSST and MNE to somatosensory MEG. We also found that peaks in MEG were mainly generated from these sources as shown in Fig. 1 and Fig. 2. Here, since no significant changes were observed in the post-stimulus interval after 320 ms, only source time courses in the post-stimulus from 0 to 320 were analysed. The validity of our approach is substantiated by three independent bits of evidence. First, the fitted dipoles explained the scalp distribution of MEG with RV of 5.5% and 10.8 ± 4.2% at the group level and single-subject level, respectively. Second, as shown in Fig. 2 the sources explaining the peak at 71 ms are mainly located in SI-l and SII-l, and the sources explaining the peak at 201 ms are mainly located in SII-r. Third, the results of source modelling are consistent with a large number of previous studies^2^,^9^,^45^.

Tactile events are accompanied by transient changes in mu (8-15 Hz) and beta (15-30 Hz) band oscillatory activity^46^,^47^. The time–varying effective connectivity shows patterns in exactly those frequency ranges. Note that there is no restriction to a frequency or frequency band imposed by the analysis technique. We therefore considered the frequency band from 5 Hz to 30 Hz for the connectivity analysis; both, previous findings about the tactile events and our analysis suggest this as the optimal frequency band.

In MEG studies of the somatosensory cortex, the tactile pulses elicit evoked responses within 50–80 ms originating from contralateral SI^48^,^49^,^50^,^51^. A study recently reported that contralateral SI was the earliest source activated by somatosensory stimuli with peaks at 63 ± 14 ms^6^. In our study, the initial response to the tactile stimulus was observed at around 70 ms, which is consistent with those previous findings. We also found that contralateral SI was the earliest activated source in response to somatosensory stimuli and that the connectivity from contralateral SI represented the earliest information flow (onset at about 20 ms to contralateral SII), which is in agreement with many previous studies^6^,^7^,^52^,^53^,^54^,^55^. Unilateral sensory stimuli are transmitted to contralateral SI from primary afferent fibers of dorsal root ganglion or trigeminal sensory neurons, also supporting our results^56^. Contralateral SII was activated after receiving somatosensory information from contralateral SI, which is consistent with a large number of studies^6^,^9^,^13^,^27^. Our results demonstrate that ipsilateral SII was activated after receiving somatosensory information from contralateral SI (onset at 20 ms) and then from contralateral SII (onset at 220 ms)^6^,^9^. There is considerable anatomical and electrophysiological evidence for interhemispheric connections between the somatosensory cortices via the corpus callosum. In particular the majority of SII neurons display bilateral receptive fields^56^,^57^,^58^. The activation of ipsilateral SII in our study is in accord with previous studies^9^,^59^. Our results indicate that functional information flow follows these anatomical pathways from contralateral SI to bilateral SII, from contralateral SII to SI, and finally from contralateral SII to ipsilateral SII in the somatosensory information processing for healthy subjects.

In conclusion, a combination of renormalized partial directed coherence and state space modelling has been demonstrated to provide a reasonable estimation of time-varying effective connectivity among neural sources on the order of milliseconds from multi-channel MEG recordings. We showed that neural sources that responded to somatosensory stimuli displayed a complex pattern of connectivity that included both feedforward and feedback information flow consistent with current views of even simple sensory responses invoking a network of activity as opposed to sequential hierarchical activation of cortical regions. Our results confirmed that this analysis approach provides somatosensory network connectivity patterns consistent with previously reported anatomical and neurophysiological measures. Our empirical validation of this novel method provides feasibility for applying this method to clinical populations to identify biomarkers of disrupted network connectivity that represent the transitions from normal state to disease state, which is crucial for understanding the mechanism of numerous diseases.

## Materials and methods

### Human somatosensory MEG study

The human somatosensory evoked MEG responses were collected as a part of a separate study. Prior to the study, the written informed consent was provided by each subject. The experimental protocols were approved by the Human Research Review Committee at University of New Mexico Health Sciences Center, Albuquerque, New Mexico. The methods were carried out in accordance with the approved guidelines. The MEG data from twenty-one healthy subjects (12 male, 9 female), aged from 12 to 20 years (15.2 ± 2.6, age ± SD), were collected in a magnetically shielded room (Vacuumschmelze – Ak3B) at the Mind Research Network in Albuquerque using a 306-channel whole-head MEG system (Elekta Neuromag) with a sampling rate of 1000 Hz and a band pass filter between 0.1-330 Hz to avoid drifts and aliasing. Prior to the experiment, fiducial points (left and right preauricular points and nasion) and head shape data were collected and checked by the Polaris system. Participants sat upright during the task and were monitored at all times by an audio and video link between the magnetically shielded room and control room. The 306-sensor MEG system measured the magnetic field distribution around the whole head of the seated subjects. The system was fully equipped with real-time motion correction and artifact rejection software. As long as the subject’s head remained within the MEG helmet, movement could be corrected to an optimal head location using the movement correction algorithm provided with the Neuromag software package. This motion corrected dataset could then be further analysed in our study without concern of subjects’ movement across epochs. The stimuli were presented while the subjects were quietly sitting with the head situated in the MEG helmet. The somatosensory stimuli were generated by allowing a short pulse of compressed air to fill an air bladder that was attached to the subjects’ right index finger. The air pulse was controlled by the Presentation software and a compressed air regulator (located outside the shielded room). The air puff stimulus lasted 50 ms with an ISI of 1.0–1.4 seconds. The air bladder pressure applied to the index finger was monitored and recorded simultaneously with MEG collection for offline analysis and interpretation of the results. We collected 120 to 180 trials for the right index finger of each subject.

The 60 Hz powerline noise was removed^60^. Raw MEG data were filtered for noise sources such as eye blinks and excessive movement, then corrected for head motion using the Neuromag MaxFilter software^61^. Heartbeat artifacts were then removed by projecting electrocardiogram (ECG) data from MEG sensor waveforms using the signal-space projection (SSP) method^62^. The data for each stimulus condition were obtained from 100 ms prior to the onset of the stimulus to 600 ms following stimulus onset. The data were baseline-corrected and subjected to a 50 Hz low-pass filter during signal processing.

### Structural MRI data acquisition

Structural MRIs were obtained for use in mapping source locations from all the subjects. Sagittal T1-weighted anatomical MR images were collected using a Siemens TIM Trio 3 Tesla MRI system with a multiecho 3D MPRAGE sequence [TR/TE/TI = 2530/1.64, 3.5, 5.36, 7.22, 9.08/1200 ms, flip ange = 7°, field of view (FOV) = 256 mm*256 mm, matrix = 256*256, 1 mm thick slice, 192 slices, GRAPPA acceleration factor = 2].

### Source time courses calculation

Sources were localised for each subject using cortical-start spatiotemporal (CSST) multidipole analysis with integrated Multiple Signal Classification (MUSIC)^63^. CSST is an objective multidipole, multistart procedure in which initial dipole locations are randomly selected from a predefined cortical volume and a nonlinear simplex search is performed for each of these initial configurations. Initial dipole locations were selected from within a predefined head volume, which was defined by a subsample of points taken from within the cortical volume, as determined by coregistered structural MRI. The error is minimized using a reduced chi-square statistic to obtain a final set of dipole configurations which most fully explain the data^63^. CSST source localization was calculated using 3,4,5,6, and 7 dipole models, based on the averaged responses occurring between 0 ms and 320 ms after the onset of the stimulus for responses to somatosensory stimuli. A shorter time window after stimuli was used for dipole modeling of somatosensory responses to increase the power in order to detect somatosensory evoked sources. The Nelder-Meade minimization procedure was carried out 1500 to 8000 times, depending on the number of dipoles in the model, to help to ensure that the procedure would reach a global minimum. The dipole model that best explained the data was selected for source time course analysis.

Following the selection of the optimal source model, the single-trial waveforms of each dipolar source were calculated within a realistic head model with the minimum norm estimate (MNE) software^64^. The inverse solution yielded estimates of continuous time series of cortical currents. For each patient, the realistic cortical surface and three layers (inner skull, outer skull and skin) were reconstructed from the anatomical MRI images using the Freesurfer software (Compumedics, Charlotte, NC). The boundary element model (BEM) was then constructed with the reconstructed surfaces. The co-registration of MEG and MRI images was achieved by matching the recorded positions of three fiducial points (nasion, left and right preauricular points) with the locations of these points from the MRI images. The lead field matrix relating MEG sensors to the cortical distributed dipoles was computed with the BEM model using MNE. The dipole model, cortical surface and lead field matrix were then used in the MNE software to extract the single-trial timecourses of sources. MNE was applied before time-frequency decomposition here.

### Renormalized partial directed coherence combined with state space modelling

Here we briefly introduce the time-dependent Granger causal connectivity analysis method employed in this study. A time continuous multivariate dynamical process *Z*(*t*) can only be observed as a multivariate, time discrete sampled signal^35^


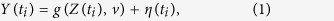


where *g*(⋅) denotes the observation function with parameter set *v*; *η*(*t*) is a Gaussian distributed independent measurement noise with a given variance. Assuming a linear observation function, we obtain the following model:


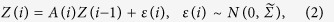



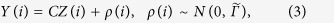


for some appropriately chosen variances 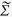 and 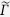 that are optimally determined in the estimation process and where *C* represents the linear observation matrix. A reasonable assumption is that the parameter matrix *A*(*i*) should change more slowly than the (stochastic) dynamics itself. The model is then augmented to the over-arching state space model as following:


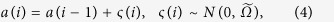



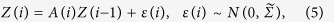



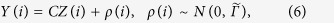


The *a*(*i*) are the matrix entries of *A*(*i*) rearranged into a vector. The causal influences can be represented as directed edges in a network, in which the nodes represent the processes. Thus the matrix *A*(*i*) contains the interactions between the components of the original process *Z*(*t*). The information about the network structure is also contained in this matrix. Since we do not make any assumption about the origin of *Z*(*t*), it can model the sensor space as well as the source space equally well.

In networks, influences with a certain delay are typically relevant. This can be accounted for in state space modelling by including previous time steps,


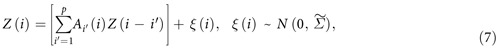


up to a maximum time lag *p*. This maximum *p* can be determined relying on a priori knowledge or based on model selection criteria, such as Akaike’s Information Criterion (AIC) used in our study^35^,^36^. The higher order process can be rewritten as a first order process by introducing


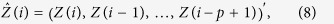



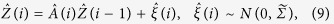


The matrix 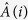 assumes the specific structure


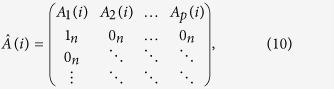


In the above form, the trajectory of the *Z*(*i*) and the *a*(*i*) can be determined purely based on the observations *Y*(*i*). The dual Kalman filters rely on the knowledge of the exact dynamics, i.e., a precise knowledge of *C*, 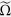, 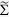, and 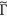
estimated by expectation maximisation algorithm^33^,^36^. The expectation maximization algorithm is an iterative algorithm that converges in the sense of maximum likelihood to the best estimator of the underlying dynamical process *Z*(*i*) and the parameters *a*(*i*)^65^.

The renormalized partial directed coherence (rPDC) is derived as follows^66^:


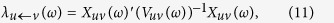


This is a frequency domain measure for Granger–causality^66^ that quantifies the direction and the strengths of network connections. *X*_*uv*_(*ω*) = [*R*(*FT*(*A*_*i*′,*uv*_)), *I*(*FT*(*A*_*i*′,*uv*_))]^′^ with R the real and I the imaginary parts, FT the Fourier transform, and *N* the number of data points analysed. The normalization by (*V*_*uv*_(*ω*)) ^− 1^ is given by the inverse of N-times the covariance matrix


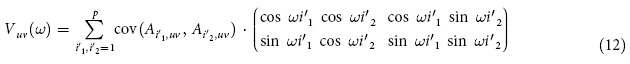


of the estimates of *X*. The covariance matrix of the estimated parameters 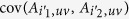 is determined in the expectation maximization algorithm. The mathematical details were demonstrated in our previous work^35^,^36^.

### Statistical analysis

In order to test whether the rPDC within the stimulation period was significantly different from the reference period, two populations were extracted for comparison: the collection of rPDC for the somatosensory stimulation period *λ*_*s*_(*n*,*f*) at each time-frequency point (*n*,*f*) from 0 to 320 ms with a sampling rate of 1000 Hz and the reference population *λ*_*R*_(*τ*,*f*|*τ*∈reference period) from -100 to 0 ms for 21 subjects. The null hypothesis is that there is no difference between the means of the two populations.

## Author Contributions

B.S., C.G., J.W. and D.L. formulated the idea of the paper and supervised the research. J.S. and T.Z. provided the MEG data samples, and made useful suggestions for the study. B.C. preprocessed the data. L.G. processed and analyzed the data. L.S., B.S. and B.C. helped the data analysis. B.S. and L.S. constructed the computational method. L.G. and L.S. wrote the manuscript. C.G., B.S. and J.S. revised the manuscript.

## Additional Information

**How to cite this article**: Gao, L. *et al*. Granger causal time-dependent source connectivity in the somatosensory network. *Sci. Rep.* **5**, 10399; doi: 10.1038/srep10399 (2015).

## Figures and Tables

**Table 1:** Source distribution of right somatosensory MEG.

**Source location**	**The number of subjects**
Left primary somatosensory cortex	21
Right secondary somatosensory cortex	17
Left secondary somatosensory cortex	15
Left secondary auditory cortex	5
Right primary somatosensory cortex	5
Anterior cingulate cortex	2
Left supramarginal gyrus	2
Right premotor cortex	1
Right supramarginal gyrus	1
Colliculus	1
Thalamus	1
Left intraparietal sulcus	1
Medial prefrontal cortex	1
Right secondary auditory cortex	1
Left primary auditory cortex	1
Posterior cingulate gyrus	1

**Table 2:** The mean and standard deviation (STD) of rPDC between SI-l, SII-l and SII-r of right somatosensory MEG responses of identified time-frequency regions in Fig. 3.

**Source**	**Mean**		**STD**
from SI-l to SII-l	0.17		0.01
from SII-l to SI-l	0.18		0.01
from SI-l to SII-r	0.16		0.01
from SII-r to SI-l	0.00		0.00
from SII-l to SII-r	0.17		0.02
from SII-r to SII-l	0.00		0.00

## References

[b1] Downar J., Crawley A. P., Mikulis D. J. & Davis K. D. A multimodal cortical network for the detection of changes in the sensory environment. Nat. Neurosci. 3, 277–283 (2000). 10700261 10.1038/72991

[b2] Mouraux A. & Iannetti G. D. Nociceptive laser-evoked brain potentials do not reflect nociceptive-specific neural activity. J. Neurophysiol. 101, 3258–3269 (2009). 19339457 10.1152/jn.91181.2008

[b3] Mouraux A., Diukova A., Lee M. C., Wise R. G. & Iannetti G. D. A multisensory investigation of the functional significance of the “pain matrix”. Neuroimage 54, 2237–2249 (2011). 20932917 10.1016/j.neuroimage.2010.09.084

[b4] Yamashiro K. *et al.* Somatosensory off-response in humans: an ERP study. Exp. Brain Res. 190, 207–213 (2008). 18584160 10.1007/s00221-008-1468-8

[b5] Schoffelen J. M. & Gross J. Source connectivity analysis with MEG and EEG. Hum. Brain Mapp. 30, 1857–1865 (2009). 19235884 10.1002/hbm.20745 PMC6870611

[b6] Hu L., Zhang Z. G. & Hu Y. A time-varying source connectivity approach to reveal human somatosensory information processing. NeuroImage 62, 217–228 (2012). 22580382 10.1016/j.neuroimage.2012.03.094

[b7] Inui K., Wang X., Tamura Y., Kaneoke Y. & Kakigi R. Serial processing in the human somatosensory system. Cereb. Cortex 14, 851–857 (2004). 15054058 10.1093/cercor/bhh043

[b8] Pons T. P., Garraghty P. E. & Mishkin M. Serial and parallel processing of tactual information in somatosensory cortex of rhesus monkeys. J. Neurophysiol. 68, 518–527 (1992). 1527572 10.1152/jn.1992.68.2.518

[b9] Blatow M., Nennig E., Durst A., Sartor K. & Stippich C. fMRI reflects functional connectivity of human somatosensory cortex. NeuroImage 37, 927–936 (2007). 17629500 10.1016/j.neuroimage.2007.05.038

[b10] Chakrabarti S. & Alloway K. D. Differential origin of projections from SI barrel cortex to the whisker representations in SII and MI. J. Comp. Neurol. 498, 624–636 (2006). 16917827 10.1002/cne.21052

[b11] Zhang H. Q. *et al.* Functional characteristics of the parallel SI- and SII-projecting neurons of the thalamic ventral posterior nucleus in the marmoset. J. Neurophysiol. 85, 1805–1822 (2001). 11352998 10.1152/jn.2001.85.5.1805

[b12] Sutherland M. T. & Tang A. C. Reliable detection of bilateral activation in human primary somatosensory cortex by unilateral median nerve stimulation. NeuroImage 33, 1042–1054 (2006). 16997579 10.1016/j.neuroimage.2006.08.015

[b13] Hari R. *et al.* Functional organization of the human first and second somatosensory cortices: a neuromagnetic study. Eur. J. Neurosci. 5, 724–734 (1993). 8261143 10.1111/j.1460-9568.1993.tb00536.x

[b14] Mima T., Nagamine T., Nakamura K. & Shibasaki H. Attention modulates both primary and second somatosensory cortical activities in humans: a magnetoencephalographic study. J. Neurophysiol. 80, 2215–2221 (1998). 9772274 10.1152/jn.1998.80.4.2215

[b15] Maldjian J. A. *et al.* Mapping of secondary somatosensory cortex activation induced by vibrational stimulation: an fMRI study. Brain Res. 824, 291–295 (1999). 10196461 10.1016/s0006-8993(99)01126-9

[b16] Wegner K., Forss N. & Salenius S. Characteristics of the human contra-versus ipsilateral SII cortex. Clin. Neurophysiol. 111, 894–900 (2000). 10802461 10.1016/s1388-2457(99)00319-3

[b17] Backes W. H., Mess W. H., van Kranen-Mastenbroek V. & Reulen J. P. Somatosensory cortex responses to median nerve stimulation: fMRI effects of current amplitude and selective attention. Clin. Neurophysiol. 111, 1738–1744 (2000). 11018487 10.1016/s1388-2457(00)00420-x

[b18] Del Gratta C. *et al.* Topographic organization of the human primary and secondary somatosensory cortices: comparison of fMRI and MEG finds. NeuroImage 17, 1373–1383 (2002). 12414277 10.1006/nimg.2002.1253

[b19] Lin Y. Y. & Forss N. Functional characterization of human second somatosensory cortex by magnetoencephalography. Behav. Brain Res. 135, 141–145 (2002). 12356444 10.1016/s0166-4328(02)00143-2

[b20] Iannetti G. D. *et al.* Representation of different trigeminal divisions within the primary and secondary human somatosensory cortex. NeuroImage 19, 906–912 (2003). 12880819 10.1016/s1053-8119(03)00139-3

[b21] Stippich C., Romanowski A., Nennig E., Kress B. & Sartor K. Time-efficient localization of the human secondary somatosensory cortex by functional magnetic resonance imaging. Neurosci. Lett. 381, 264–268 (2005). 15896481 10.1016/j.neulet.2005.02.004

[b22] Korvenoja A. *et al.* Activation of multiple cortical areas in response to somatosensory stimulation: combined magnetoencephalographic and functional magnetic resonance imaging. Hum. Brain Mapp. 8, 13–27 (1999). 10432179 10.1002/(SICI)1097-0193(1999)8:1<13::AID-HBM2>3.0.CO;2-B PMC6873291

[b23] Tan H. R., Wuhle A. & Braun C. Unilaterally applied stimuli in a frequency discrimination task are represented bilaterally in primary somatosensory cortex. Neurol. Clin. Neurophysiol. 2004, 83 (2004). 16012597

[b24] Nihashi T. *et al.* Contralateral and ipsilateral response in primary somatosensory cortex following electrical median nerve stimulation - an fMRI study. Clin. Neurophysiol. 116, 842–848 (2005). 15792893 10.1016/j.clinph.2004.10.011

[b25] Nevalainen P., Ramstad R., Isotalo E., Haapanen M. L. & Lauronen L. Trigeminal somatosensory evoked magnetic fields to tactile stimulation. Clin. Neurophysiol. 117, 2007–2015 (2006). 16859989 10.1016/j.clinph.2006.05.019

[b26] Hlushchuk Y. & Hari R. Transient suppression of ipsilateral primary somatosensory cortex during tactile finger stimulation. J. Neurosci. 26, 5819–5824 (2006). 16723540 10.1523/JNEUROSCI.5536-05.2006 PMC6675271

[b27] Iwamura Y. Hierarchical somatosensory processing. Curr. Opin. Neurobiol. 8, 522–528 (1998). 9751655 10.1016/s0959-4388(98)80041-x

[b28] Winterhalder M. *et al.* Comparison of linear signal processing techniques to infer directed interactions in multivariate neural systems. Sig. Proc. 85, 2137–2160 (2005).

[b29] Hemmelmann D. *et al.* Modelling and analysis of time-variant directed interrelations between brain regions based on BOLD-signals. NeuroImage 45, 722–737 (2009). 19280694 10.1016/j.neuroimage.2008.12.065

[b30] Andrzejak R. G., Ledberg A. & Deco G. Detecting event-related time-dependent directional couplings. New J. Phys. 8, 6 (2006).

[b31] Martini M., Kranz T. A., Wagner T. & Lehnertz T. Inferring directional interactions from transient signals with symbolic transfer entropy. Phys. Rev. E 83, 011919 (2011). 10.1103/PhysRevE.83.011919 21405725

[b32] Harvey A. C. Forecasting, Structural Time Series Models and the Kalman Filter. Cambridge University Press (1994).

[b33] Shumway R. H. & Stoffer D. S. Time Series Analysis and Its Applications. New York: Springer (2000).

[b34] Hamilton J. D. Time series analysis. Princeton University Press (1994).

[b35] Schelter B. *et al.* Overarching framework for data-based modelling. European Phys. Lett. 83, 30004 (2014).

[b36] Sommerlade L. *et al.* Inference of Granger causal time-dependent influences in noisy multivariate time series. J. Neurosci. Methods 203, 173–185 (2012). 21944999 10.1016/j.jneumeth.2011.08.042

[b37] Buzsáki G. & Draguhn A. Neuronal oscillations in cortical networks. Science 304, 1926–1929 (2004). 15218136 10.1126/science.1099745

[b38] Schnitzler A. & Gross J. Normal and pathological oscillatory communication in the brain. Nat. Rev. Neurosci. 6, 285–296 (2005). 15803160 10.1038/nrn1650

[b39] Varela F., Lachaux J. P., Rodriguez E. & Martinerie J. The brainweb: Phase synchronization and large-scale integration. Nat. Rev. Neurosci. 2, 229–239 (2001). 11283746 10.1038/35067550

[b40] Granger C. W. J. Investigating causal relations by econometric models and cross spectral methods. Econometrica 37, 424–438 (1969).

[b41] Baccala L. A. & Sameshima K. Partial directed coherence: a new concept in neural structure determination. Biol. Cybern. 84, 463–474 (2001). 11417058 10.1007/PL00007990

[b42] Pereda E., Quiroga R. Q. & Bhattacharya J. Nonlinear multivariate analysis of neurophysiological signals. Prog. Neurobiol. 77, 1–37 (2005). 16289760 10.1016/j.pneurobio.2005.10.003

[b43] Nolte G. *et al.* Identifying true brain interaction from EEG data using the imaginary part of coherence. Clin. Neurophysiol. 115, 2292–2307 (2004). 15351371 10.1016/j.clinph.2004.04.029

[b44] Gao L., Zhang T. S., Wang J. & Stephen J. Facilitating neuronal connectivity analysis of evoked responses by exposing local activity with principal component analysis preprocessing: simulation of evoked MEG. Brain Topogr. 26, 201–211 (2013). 22918837 10.1007/s10548-012-0250-1 PMC3993084

[b45] Shimojo M., Svensson P., Arendt-Nielsen L. & Chen A. C. Dynamic brain topography of somatosensory evoked potentials and equivalent dipoles in response to graded painful skin and muscle stimulation. Brain Topogr. 13, 43–58 (2000). 11073093 10.1023/a:1007834319135

[b46] Cheyne D., *et al.* Neuromagnetic imaging of cortical oscillations accompanying tactile stimulation. Cognitive Brain Research 17(3), 599–611 (2003). 14561448 10.1016/s0926-6410(03)00173-3

[b47] Spitzer B. & Blankenburg F. Stimulus-dependent EEG activity reflects internal updating of tactile working memory in humans. Proceedings of the National Academy of Sciences of the United States of America 108(20), 8444–8449 (2011). 21536865 10.1073/pnas.1104189108 PMC3100957

[b48] Elbert T., Junghofer M., Scholz B. & Schneider S. The separation of overlapping neuromagnetic sources in first and secondary somatosensory cortices. Brain Topogr. 7, 275–282 (1995). 7577325 10.1007/BF01195253

[b49] Mauguiere F. *et al.* Activation of a distributed somatosensory cortical network in the human brain. A dipole modelling study of magnetic fields evoked by median nerve stimulation. Part I: location and activation timing of SEF sources. Electroencephalogr. Clin. Neurophysiol. 104, 281–289 (1997). 9246065 10.1016/s0013-4694(97)00006-0

[b50] Simoes C. *et al.* Functional overlap of finger representations in human SI and SII cortices. J. Neurophysiol. 86, 1661–1665 (2001). 11600629 10.1152/jn.2001.86.4.1661

[b51] Tuunanen P. *et al.* Comparison of BOLD fMRI and MEG characteristics to vibrotactile stimulation. NeuroImage 19, 1778–1786 (2003). 12948732 10.1016/s1053-8119(03)00256-8

[b52] Van de Wassenberg W., Van der Hoeven J., Leenders K. & Maurits N. Multichannel recording of median nerve somatosensory evoked potentials. Neurophysiol. Clin. 38, 9–21 (2008). 18329546 10.1016/j.neucli.2007.08.002

[b53] Kandel E. R., Schwartz J. H. & Jessell T. M. Principles of Neural Science. McGraw-Hill Health Professions Division, New York (2000).

[b54] Allison T. *et al.* Human cortical potentials evoked by stimulation of the median nerve. I. Cytoarchitectonic areas generating short-latency activity. J. Neurophysiol. 62, 694–710 (1989a). 2769354 10.1152/jn.1989.62.3.694

[b55] Allison T., McCarthy G., Wood C. C., Williamson P. D. & Spencer D. D. Human cortical potentials evoked by stimulation of the median nerve. II. Cytoarchitectonic areas generating long-latency activity. J. Neurophysiol. 62, 711–722 (1989b). 2769355 10.1152/jn.1989.62.3.711

[b56] Jones E. G. & Peters A. Cerebral Cortex. New York: Plenum Press (1986).

[b57] Killackey H. P., Gould H. J. III, Cusick C. G., Pons T. P. & Kaas J. H. The relation of corpus callosum connections to architectonic fields and body surface maps in sensorimotor cortex of new and old world monkeys. J. Comp. Neurol. 219, 384–419 (1983). 6643713 10.1002/cne.902190403

[b58] Innocenti G. M. General organization of callosal connections in the cerebral cortex. In Jones E. G. & Peters A. (Eds.) Cerebral Cortex, Sensory-Motor Areas and Aspects of Cortical Connectivity. New York: Plenum Press 5, 291–353 (1986).

[b59] Shuler M. G., Krupa D. J. & Nicolelis M. A. Bilateral integration of whisker information in the primary somatosensory cortex of rats. J. Neurosci. 21, 5251–5261 (2001). 11438600 10.1523/JNEUROSCI.21-14-05251.2001 PMC6762838

[b60] Zhang T. & Okada Y. Recursive artifact windowed-single tone extraction method (RAW-STEM) as periodic noise filter for electrophysiological signals with interfering transients. J. Neurosci. Methods 155, 308–318 (2006). 16466806 10.1016/j.jneumeth.2005.12.022

[b61] Taulu S. & Kajola M. Presentation of electromagnetic multichannel data: The signal space separation method. J. Appl. Phys. 97, 124905 (2005).

[b62] Uusitalo M. A. & Ilmoniemi R. J. Signal-space projection method for separating MEG or EEG into components. Med. Biol. Eng. Comp. 35, 135–140 (1997). 10.1007/BF02534144 9136207

[b63] Ranken D. M., Stephen J. M. & George J. S. MUSIC seeded multi-dipole MEG modeling using the constrained start spatio-temporal modeling procedure. Neurol. Clin. Neurophysiol. 2004, 80 (2004). 16012631

[b64] Hämäläinen M. S. & Ilmoniemi R. J. Interpreting magnetic-fields of the brain-minimum norm estimates. Med. Biol. Eng. Comp. 32, 35–42 (1994). 10.1007/BF02512476 8182960

[b65] Honerkamp J. Statistical Physics. Springer (2012).

[b66] Schelter B., Timmer J. & Eichler M. Assessing the strength of directed influences among neural signals using renormalized partial directed coherence. J. Neurosci. Methods 179, 121–130 (2009). 19428518 10.1016/j.jneumeth.2009.01.006

